# Individualised radial extracorporeal shock wave therapy (rESWT) for symptomatic calcific shoulder tendinopathy: a retrospective clinical study

**DOI:** 10.1186/s12891-017-1873-x

**Published:** 2017-12-06

**Authors:** Nikolaos Malliaropoulos, Dawn Thompson, Maria Meke, Debasish Pyne, Dimosthenis Alaseirlis, Henry Atkinson, Vasileios Korakakis, Heinz Lohrer

**Affiliations:** 1 Sports and Exercise Medicine Clinic, Asklipiou 17, 54639 Thessaloniki, Greece; 2 National Track and Field Centre, Sports Medicine Clinic of S.E.G.A.S, Thessaloniki, Greece; 3 European Sports Care, Harley Street, London, W1G UK; 4 Sports Clinic, Rheumatology Department, Barts Health NHS Trust, Bancroft Road, London, E1 4DG UK; 5 Centre for Sports & Exercise Medicine, Queen Mary, University of London, Bancroft Road, London, E1 4DG UK; 6 Imperial College School of Medicine, Imperial College, London, UK; 7 Department of Orthopaedics, General Clinic Euromedica, Thessaloniki, Greece; 8 London Sports Orthopaedics, Bridge Hospital, 27 Tooley St, London, SE1 2PR UK; 9 North Middlesex University Hospital, Sterling Way, London, N18 1QX UK; 10 Aspetar, Orthopaedic and Sports Medicine Hospital, Doha, Qatar; 11 Faculty of Sport Science and Physical Education, University of Thessaly, Trikala, Greece; 12 HOMTD, Hellenic Orthopaedic Manipulative Therapy Diploma, Athens, Greece; 13 European SportsCare Network (ESN) - Zentrum für Sportorthopädie, Borsigstrasse 2, 65205 Wiesbaden, Nordenstadt Germany; 14 Institute for Sport and Sport Sciences, Albert-Ludwigs-Universität Freiburg i. Brsg, Schwarzwaldstraße 175, 79117 Freiburg, Germany

**Keywords:** ESWT, Radial extracorporeal shockwave therapy, Recurrence rate, Symptomatic shoulder calcifying tendinopathy, Treatment

## Abstract

**Background:**

A retrospective single centre cohort analysis was performed to evaluate an individualised radial extracorporeal shock wave therapy (rESWT) protocol for treatment of symptomatic calcific shoulder tendinopathy.

**Methods:**

67 patients (79 Shoulders) were identified with 76 shoulders included for analysis. rESWT treatment protocol was adapted according to individual response to treatment. Variables included number of sessions, shockwave impulses, pressure and frequency. Success rate was estimated as the percentage of patients having ≥60% visual analogue score (VAS) pain decrease at follow-up. Recurrence at 1 year was recorded.

**Results:**

Using this individualised symptom guided protocol, patients underwent a mean of 7 ± 1.5 rESWT sessions, with mean pressure of 1.7 ± 0.2 bar, mean frequency of 5 ± 0.3 Hz and 2175 ± 266 impulses. The mean pre-treatment VAS score of 6.7 ± 1.1 was significantly decreased to 3.2 ± 0.8 immediately post-treatment, 2.6 ± 0.9 at 1 month, 1.7 ± 1.0 at 3 months and 0.8 ± 1.0 at 1 year follow up (α = 0.05). One-year success rate was estimated at 92% and 1-year recurrence rate was 7%.

**Conclusions:**

We conclude that in this retrospective study an individualised rESWT protocol resulted in a high success rate with low number of recurrences. Randomised controlled trials to support these findings are recommended.

## Background

Symptomatic calcifying tendinopathy of the shoulder is a common condition characterised by the accumulation of calcium hydroxyapatite crystals within the tendons of the rotator cuff muscles [1].

The condition often presents acutely, though can be sub-acute or chronic [2]. The natural history of the disease is unclear, some consider it to be self-limiting though others report a protracted clinical course resulting in significant disability [3, 4]. Calcification is most commonly found within the proximal supraspinatous tendon at the ‘critical zone,’ an area of tendon at risk of tissue hypoxia [5–8]. The degenerative process may occur due to an impingement syndrome, poor blood supply, increased stress on the tendon and/or anatomical variations of the acromion [4, 9]. Typical features of symptomatic calcific shoulder tendinopathy include pain and temporary loss of function which can be severe and is worse during shoulder abduction [8, 10]. Clinical findings often include local tenderness usually with mechanical block and occasional crepitus; radiographically detectable calcification near the rotator cuff insertion is also seen [11, 12].

During the acute phase, lasting 3 weeks - 6 months, pain is severe due to an aggressive inflammatory response. The mainstay of treatment in the acute painful phase revolves around pain management and the maintenance of upper limb function; this may be achieved with non-steroidal anti-inflammatories, physiotherapy and therapeutic ultrasound. Other treatment options include transcutaneous electrical nerve stimulation (TENS) and needle aspiration [13, 14].

In cases where conservative treatment has not resulted in a significant reduction of symptoms, the use of extracorporeal shock wave therapy (ESWT) has been gaining in popularity as an alternative to surgical excision of the calcifications [9]. ESWT can be used to deliver low or high energy shock waves to a specific target within the body. The shockwave acts specifically at the calcific/soft tissue interface (high acoustic impedance). Biologic effects are disruption of fibrous tissue resulting in revascularisation and healing, together with changes in sensory processing of pain [9].

An alternative to ESWT is radial shock wave therapy (rESWT) in which pressure waves are generated and transmitted in a radial fashion to meet the target zone [15]. In rESWT the focal point is upon the tip of the applicator compared to ESWT in which the focal point is deeper and inside the body. It has been suggested that rESWT as compared to ESWT may be less painful and is more likely to include the target zone within the wave propagation [15]. rESWT has been shown to be effective in treatment of musculoskeletal conditions such as plantar fasciitis, epicondylitis of the elbow and finger flexor tenosynovitis [16–19].

To date there are limited one year follow up results for rESWT in the treatment of calcifying shoulder tendinopathy. Most studies have used a ‘one size fits all’ approach to treatment with all patients receiving a standardised and therefore identical treatment regime. The aim of our study was to retrospectively evaluate the effectiveness of an individualised rESWT treatment protocol with a 12 month follow up.

## Methods

### Study design

The study was carried out retrospectively as an uncontrolled study on a cohort of patients sequentially diagnosed with symptomatic calcifying shoulder tendinopathy at a secondary care Musculoskeletal Sports Medicine Clinic based in Thessaloniki, Greece from 2006 to 2013. The diagnosis of symptomatic calcifying shoulder tendinopathy was made through a combination of detailed history, clinical examination and ultrasound scanning. Ultrasound scanning was used as opposed to shoulder radiograph to allow rapid diagnosis on day of presentation, detailed assessment of rotator cuff and accurate pre-treatment localisation of calcification. Following diagnosis, the treatment options were discussed with the patients including the use of rESWT.

67 patients (79 shoulders) were initiated on the rESWT treatment after being diagnosed with calcifying shoulder tendinopathy. Three of the patients quit the treatment protocol before completion, one for financial reasons and two due to time constraints.

Inclusion and Exclusion criteria are shown in Table 1.

**Table 1:** Inclusion and Exclusion Criteria

Exclusion Criteria	Inclusion Criteria
Patients with acute (severely painful) calcific shoulder tendinopathy	Patients with symptomatic calcific shoulder tendinopathy
Patients with rotator cuff rupture	Age greater than 18
History of previous surgery or malignancy	Every session performed was under ultrasound guidance.
Corticosteroid injection within the preceding 6 weeks	
Previous ESWT treatment	

### Patient population

64 patients (76 shoulders) were included. The series comprised 25 (39%) men and 39 (61%) women, with a mean age of 45 ± 8, (26–63 years old). The mean pre-treatment pain duration was 12.5 ± 19 months (1–96 months), median pain duration was 6 months. Detailed patient demographics can be seen in Table 2.

**Table 2:** Characteristics of the patients

Characteristic	N (Percentage)
**Sex**	
Male	25 (39.1%)
Female	39 (60.9%)
**Age**	
mean (SD)	44.7 (8.1) Years
**Shoulder affected**	
Right	19 (25%)
Left	33 (43%)
Bilateral	12 (32%)
**Pre-treatment pain duration**	
< 3 months	27 (35.5%)
3–6 months	16 (21.1%)
6–12 months	20 (26.3%)
12–24 months	5 (6.6%)
> 24 months	8 (10.5%)

### Treatment protocol

Prior to each treatment, a surface marking corresponding to the area of calcification was identified and marked under ultrasound guidance by the treating Consultant SEM Physician. rESWT was then applied by a trained physiotherapist using the Storz Medical Masterpuls MP 200 (Storz Medical; Tägerwilen, Switzerland) rESWT device. Ultrasound jelly was applied to the area prior to commencing treatment in order to reduce the loss of energy from the device.

Each patient received an individualised protocol based on the severity of their symptoms, their tolerance to the rESWT procedure and response to treatment [20]. This was achieved by adjusting pressure (bar), frequency (Hz) and the number of sessions. The pressure was set at a minimum of 1 bar but adjusted according to patient’s tolerance to pain. The total number of rESWT sessions varied, however the minimum was 3 sessions. RESWT sessions were stopped when visual analogue score (VAS) was less than or equal to 2 for the week prior to appointment or patients reported marked improvement in symptoms. Patients received 1 treatment per week to allow an adequate time for recovery and healing. Patients were advised against the use of non-steroidal anti inflammatories during treatment.

### Study outcomes

Four follow up intervals were considered - immediately after rESWT treatment completion, after 1 month, after 3 months and after 12 months. VAS self-evaluation scales were used to record pain intensity at each follow up time interval, patients were additionally asked to consider night pain when providing VAS score. It is accepted that VAS consists of a straight line with equal intervals of 1 cm, ranging from 0 “no pain” to 10 “worst imaginable pain” [21].

The recurrence rate was assessed at the 1 year follow up appointment. Patients were considered to have had recurrence of symptoms if additional treatment was needed due to ongoing symptoms or if they reported a VAS score greater than or equal to two.

The mean number of shockwave impulses, mean pressure and mean frequency applied were assessed retrospectively following the individualised rESWT treatment protocols. The total number of rESWT sessions in all patients were calculated.

Successful treatment was considered in those patients reporting a greater than 60% decrease in their VAS pain scores; from which the success rates were estimated for each of the follow up stages [22]. The relation between pre-treatment pain duration and the VAS outcome scores was tested, as was the relation between the pre-treatment pain duration and the number of rESWT sessions.

### Statistics

Spreadsheets and the statistical package SPSS were used for statistical analysis. The demographic, biological and clinical characteristics were analysed using descriptive statistical methods. Pre-treatment pain duration was analysed both as a scale variable and as a categorical.

Wilcoxon Signed Ranks Test and Monte Carlo simulation was used to test significant VAS score reductions at each follow up stage comparing the preceding scores and the baseline scores, with a 95% (α = 0.05) confidence level. Spearman’s rho correlation was used to explore correlations between scale variables, these were set at a 95% (α = 0.05) confidence level.

## Results

**Fig. 1:**
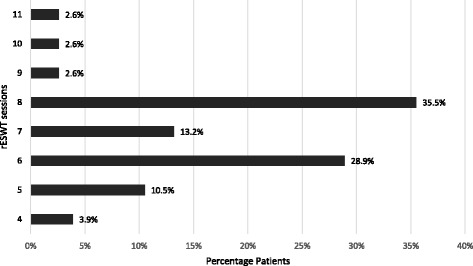
Total number of rESWT sessions applied

Of the 76 shoulders with calcific tendinopathy, the mean total number of rESWT sessions was 7 ± 1.5 (range 4–11). The majority of the shoulders received 8 rESWT sessions (27 shoulders), 6 rESWT sessions (22 shoulders) or 7 rESWT sessions (10 shoulders) (Fig. 1). There was no statistically significantly correlation between pre-treatment pain duration and number of rESWT sessions (*r* = 0.080, *p* = 0.494).

Mean pressures were 1.7 ± 0.2 bar, at a mean frequency of 5 ± 0.3 Hz with 2175 ± 266 impulses during the rESWT sessions (Table 3).

**Table 3:** Mean number pulses, mean pressure, mean frequency and number of shoulders contributing to successive sessions

rESWT session	Shoulders (N)	Pressure (bar)		Frequency (Hz)		Pulse (N)	
		mean	SD	mean	SD	mean	SD
1st	76	1.5	0.2	5	0.4	1987	114.7
2nd	76	1.5	0.2	5	0.2	2003	128.6
3rd	76	1.6	0.2	5	0.2	2054	228.9
4th	74	1.7	0.3	5	0.3	2081	243.7
5th	71	1.8	0.3	5	0.3	2114	239.8
6th	65	1.8	0.3	5	0.3	2145	271.0
7th	41	1.8	0.3	5	0.2	2195	320.9
8th	35	1.8	0.3	5	0.0	2260	341.5
9th	8	1.7	0.3	5	0.0	2425	395.5
10th	5	1.6	0.2	6	1.3	2500	353.6
11th	3	1.6	0.2	5	0.0	2167	288.7

Statistically significant decreases in VAS scores were recorded in all consecutive follow up stages, from baseline to immediately after treatment (z = −7.676, *p* = 0.000), from immediately after treatment to 1st month follow up (z = −6.152, p = 0.000), from 1st month to 3rd month follow up (z = −6.808, p = 0.000) and from 3rd month to 1 year follow up (z = −6.732, p = 0.000) (Table 4).

**Table 4:** Vas scores related samples

		Mean (VAS)	SD	Mean VAS reduction	*p* value
Pair 1 (*n* = 76)	Baseline VAS	6.7	1.1	3.4	** 0.000
	Post-treatment VAS	3.2	0.8		
Pair 2 (n = 76)	Post-treatment VAS	3.2	0.8	0.7	** 0.000
	1st month VAS	2.6	0.9		
Pair 3 (n = 76)	1st month VAS	2.6	0.9	0.9	** 0.000
	3rd month VAS	1.7	1.0		
Pair 4 (n = 76)	3rd month VAS	1.7	1.0	0.8	** 0.000
	1-year VAS	0.8	1.0		

**statistically significant difference (p = <0.001)

**Fig. 2:**
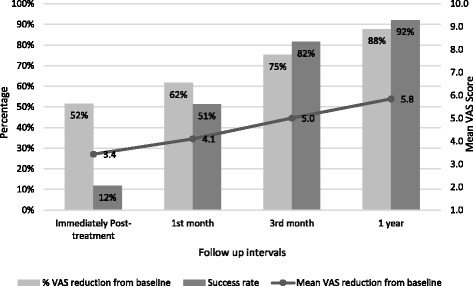
Success rates (>60% decrease in VAS score), mean and percentage VAS score reduction

VAS reductions from baseline were also statistically significant at all follow up intervals from baseline to immediately after treatment (z = −7.676, p = 0.000), to 1st month follow up (z = −7.663, p = 0.000), to 3rd month follow up (z = −7.627, p = 0.000) and 1 year (z = −7.628, p = 0.000) follow up respectively. Treatment became increasingly successful with the passage of time, starting with a 12% success rate immediately post-treatment, increasing to a 92% success rate at 1-year follow-up (Fig. 2). The 1-year recurrence rate was 7%, with 93% (71 shoulders) of the shoulders experiencing no further symptoms that required treatment.

Spearman’s rho correlation was positive and weak but remained significant between pre-treatment pain duration and post-treatment VAS scores (*r* = 0.386, *p* = 0.001), 1st month VAS score (*r* = 0.330, *p* = 0.004), 3rd month VAS score (*r* = 0.329, p = 0.004) and with 1-year VAS score (*r* = 0.349, *p* = 0.002).

Minor adverse events were recorded in 5 patients; including mild local skin irritation in three patients and post treatment discomfort lasting up to two days in two patients. No patients dropped out of the study as a direct result of adverse events. No patients reported long term use of analgesia during the study.

## Discussion

In summary this non-controlled retrospective study demonstrates a 92% success rate at 12 months follow up, with 52% reduction in mean VAS immediately post treatment, 62% at 1 month and 75% at 3 months. Importantly this improvement in symptoms was maintained at 1 year with an 88% mean VAS reduction from baseline at 12 months and only 7% rates of recurrence.

To date, limited trials have been conducted using rESWT in the treatment of calcific shoulder tendinopathy with only one randomised controlled trial found in the literature [15]. As in our study, the majority of published studies to date have been of a retrospective nature, however in contrast to our trial, the literature reports on a standardised treatment regimen [15, 23, 24]. This paper described a previously unreported “Individualised” protocol using a variable number of pulses, pressures, frequencies, and number of sessions. Each regime was designed according to the severity of the patients’ symptoms, and the patients’ tolerance and responses to the treatment.

Whilst it is difficult to make direct comparisons between studies, a comparison to the treatment arm in one randomised study using a predefined treatment protocol [15] demonstrated that the patients in our series received a lower mean pulse number (2500 pulses vs. 2175 pulses respectively), at a lower mean frequency (10 Hz vs 5 Hz respectively) and at a lower mean pressure (2.5 bar vs. 1.7 bar respectively). But at the expense of higher mean number of sessions (4 sessions vs. 7 sessions (range 4–11) respectively).

While not specifically reported, patients often find shockwave treatment quite uncomfortable. The individualised protocol utilised in this study with a lower number of pulses, a lower frequency and lower pressure may reduce patient discomfort, and allow shockwave treatment to be performed in those who find the procedure difficult to tolerate.

Despite the differences in study protocols our treatment outcomes were similar to those of previous reports [15]. In the randomised study described above patients showed significantly improved visual analog scores (VAS) at one-week post treatment, this improvement was maintained at 6 months follow up [15]. Patients in this trial had a higher pretreatment VAS score of 8 but a similar post treatment VAS of 0.9 when compared to patients in our trial (6.7 and 0.8 respectively). It would however have been interesting to see if extended follow up in this previous study would result in a higher recurrence rate [15]. Additional studies on the use of rESWT in calcific shoulder tendinopathy also used standardised, fixed treatment regimens [23, 24]. For example, one such study used a regimen of 10 Hz, 3 bar pressure and 2000 shocks for 3–5 successive treatments [23]. In this study mean pretreatment VAS of 4.7 decreased to a mean post treatment VAS of 2.4 (6 months follow up).

Thus one may infer that our customised treatment protocol has demonstrated comparable if not superior results when compared to other published material. We propose that by using an individualised protocol it is possible to tailor treatment according to patient tolerance and response to treatment and to treat each patient and their condition as an individual. Future prospective studies may wish to directly compare standardised protocol with individualised protocol in order to record long term outcome and capture data for patient tolerance to and acceptance of treatment.

Furthermore studies of rESWT in calcific shoulder tendinopathy have tended to focus on short term outcomes at 3 and 6 months follow up. This study has demonstrated further improvement in symptoms at a 1 year follow up, with a low rate of recurrence. Whilst our study is one of the first to show extended outcomes up to a year after treatment it would be interesting to further investigate recurrence rates beyond this time frame.

A lack of a control arm to allow for comparison to be made with other treatment modalities or placebo was a major limitation to this study. Systematic reviews have shown ESWT to be the most thoroughly investigated and effective treatment for calcific shoulder tendinopathy over the short to medium term with a significant improvement in pain and function compared to other interventions [25, 26]. However due to lack of published data only one randomised trial of rESWT treatment was included within this systematic review [25, 26].

A high quality randomised controlled study compared high energy ESWT to low energy ESWT and sham therapy, and found a 12-month improvement in mean VAS score of 5.6 in those receiving high energy ESWT compared to 2.6 and 1.9 in those receiving low energy ESWT and sham therapy respectively [22]. Our study found a comparable mean improvement in VAS of 5.8, however due to differences in study designs (ESWT as opposed to rESWT), primary outcomes and lack of control group, it is difficult to make any direct or meaningful comparisons.

Our study was carried out retrospectively and as such its results must be interpreted cautiously. To date only 1 randomised trial of rESWT vs. placebo exists [15]. It has been suggested that the use of rESWT may result in a higher likelihood of the target area being included in the treatment area without the need for continual ultrasound guidance [15]. Randomised controlled trials are needed to directly compare various treatment modalities including ESWT, rESWT and control or SHAM therapy to further evaluate which treatment confers the greatest benefit.

In our study, primary focus was improvement of symptoms and reduction in VAS score as opposed to radiographical resolution of calcification. Access to shoulder radiographs was not possible at the secondary care center in Thessaloniki and given that calcification could be accurately identified on ultrasonography, reduced exposure to radiation and allowed for accurate real time localisation of calcific deposits prior to individual treatment sessions, ultrasonography was used as an alternative. In addition, whilst interpretation of ultrasonography can be subjective in terms of sensitivity and specificity, this effect was minimised by all ultrasound examinations being performed by the same lead clinician with several years of experience in clinical imaging. To further investigate the effect of rESWT, studies could consider pre and post treatment shoulder radiographs to look for reduction in size of calcification.

It is known that in some individuals calcifying deposits may resolve spontaneously over time. Reports suggest between 9.3% and 33% of calcifications may disappear within 3 years. However, this process may take several years significantly impacting on quality of life in the interim [15, 27–30]. We found spearman’s rho correlation between pre-treatment pain duration and post-treatment VAS scores was positive and weak but significant, suggesting that those who had a longer pre-treatment pain duration had higher post-treatment VAS scores. This finding provides a rational for commencing treatment early as opposed to waiting to see if symptoms resolve spontaneously during the natural course of this pathology. Studies have shown higher 12-month improvements in VAS score in patients receiving ESWT therapy as opposed to SHAM therapy, suggesting that ESWT can result in symptom improvement earlier than seen in the natural disease process (22). A direct comparison of rESWT and SHAM or no therapy would help to differentiate treatment effect from placebo or natural resolution of calcifying deposits.

A number of studies of calcific shoulder tendinopathy have used the Constant Shoulder score to determine response to treatment [15, 24, 31]. This score looks at shoulder function, pain and range of motion within the preceding 4 weeks. Our study chose to use mean improvement in VAS from baseline as the main outcome measure. Whilst VAS score is a commonly utilised scoring mechanism, the use of the Constant Shoulder score in future studies, might allow one to make better comparisons with other published data.

## Conclusions

We have demonstrated high success rates and low recurrence rates at 1 year after rESWT treatment of calcific shoulder tendinopathy. Our results are comparable with data presented in the literature. Whilst the retrospective uncontrolled nature of the study should be considered we assume that by adapting treatment parameters in line with patient-guided feedback, better results can be achieved in the treatment of calcific shoulder tendinopathy. Additional prospective studies are needed in this area to gain further clarity and guide patient care.

## Abbreviations

ESWT: Extracorporeal shock wave therapy
rESWT: radial extracorporeal shock wave therapy
TENS: Transcutaneous electrical nerve stimulation
VAS: Visual Analogue Score
